# Using Humor to Promote Social Distancing on Tiktok During the COVID-19 Pandemic

**DOI:** 10.3389/fpsyg.2022.887744

**Published:** 2022-06-23

**Authors:** Yi Xiao, Shubin Yu

**Affiliations:** ^1^School of New Media and Communication, Tianjin University, Tianjin, China; ^2^Center for Crisis Management Research, Tsinghua University, Beijing, China; ^3^Department of Communication and Culture, BI Norwegian Business School, Oslo, Norway

**Keywords:** humor, health communication, COVID-19, risk perception, RAMS

## Abstract

**Background:**

During the COVID-19 pandemic, many humorous videos on how to practice social distancing appeared on social media. However, the effect of using humor as a crisis communication strategy to persuade people to conform to social distancing rules is not known.

**Objective:**

Drawing on the literature on humorous message framing and crisis communication, this research explores the effectiveness of a humorous message in communicating social distancing rules in two crisis severity phases (low vs. high severity) and also evaluates how humor affects individuals’ online and offline engagement intentions during the COVID-19 pandemic.

**Methods:**

A 2 (message framing: humorous vs. non-humorous) x 2 (crisis severity phase: low vs. high) between-subjects design experiment was conducted to test the research questions during the first weeks of the COVID-19 pandemic in China from January 30 to February 2, 2020.

**Results:**

The results showed that the severity of the phase of a health crisis can significantly affect stakeholders’ online and offline responses toward the disease. More specifically, in a low severity phase, humor led to increased source likability for the message, and more online and offline engagement intentions. However, no differences between a humorous and non-humorous message in perceived risk were observed. Whereas, in a high severity crisis phase, humor reduced individuals’ offline engagement intentions and a decrease in perceived risk, no significant difference was found between a humorous and non-humorous message on source likeability.

**Conclusion:**

Humor can motivate both more online engagement and offline protective action intention when the crisis severity phase is low, while when crisis severity soars, a non-humorous message should be more desirable. More specifically, using humor in communicating information about an infectious disease can enhance the spokesperson’s likeability in a low severity phase, and also helps to spread health information to a larger audience. While, the negative side of using humor in communicating an infectious disease appears in severe crisis phases, as it then decreased the public’s perception of risk, and triggers less protective actions. Going beyond previous research, this study recognized that crisis severity changes in different phases of the spread of infectious disease, thereby providing actionable strategy selections for crisis practitioners in a dynamic communication environment.

## Introduction

To combat the spread of the coronavirus, many village leaders in China and mayors in Italy used multiple media channels to communicate the importance of social distancing and remind citizens to stay at home. TikTok has become an emerging social media platform to communicate public health messages (Basch et al., 2020). For example, Chinese village leaders’ TikTok micro-videos and Italian mayors’ Facebook Live video clips about enforcing coronavirus quarantine rules became global viral hits. Some won unexpected celebrity status after furiously shouting at and scolding people who flouted quarantine laws in an aggressively humorous manner. Leaders revealed the most absurd stories and justifications used by citizens to explain their breaches of the rules, like playing ping-pong at the beach, pretending to go for a run, or calling hairdressers to their homes to have their hair done. For instance, a video about the mayor of Reggio Calabria told a virus-lockdown dodger that he is not a Will Smith character: “I saw a fellow citizen amiably jog up and down the street accompanied by a dog that was visibly worn out. I stopped and told him, look this is not a movie. You are not Will Smith in *I Am Legend*. Go home!” The mayor of Lucera raged at citizens calling hairdressers to their homes: “What is the damn point? Do you understand that coffins are closed? Who will see all these beautiful hairstyles in the coffins?”

The use of humor has previously been found effective in promoting health communication engagement, reducing the public’s defensive responses, and ultimately increasing the effectiveness of health information (Hendriks and Janssen, 2018). For instance, humorously framed public service announcements help motivate more cancer detection behaviors as they reduce anxiety about self-exams (Nabi, 2015). Humor also performs well in preventive health communication (e.g., regarding alcohol, tobacco, and obesity) through prolonged attention and better-recognized content (Blanc and Brigaud, 2014). When using humor in communicating climate change, a humorous appeal produces greater climate change activism intentions than a non-humorous message (Skurka et al., 2018)—though the humor was also found to decrease perceived climate change risk to humans through reduced anger and fear.

Although previous research suggested that positive emotions were important coping mechanisms during a crisis (Fredrickson et al., 2003), the effect of humorous framing during a crisis on an individual’s online and offline engagement intentions may differ. For instance, in 2011, the Center for Disease Control and Prevention in the US launched a campaign called “Preparedness 101: Zombie Apocalypse” on social media. Follow-up research revealed that humorous messages help to motivate more online engagement by quickly spreading the information, while weakening the individual’s intention to take protective action offline (Fraustino and Ma, 2015).

Using humor to communicate risk and crisis events has long been regarded as a double-edged sword because the effect varies with crisis severity. People were found less likely to engage with humorous content on Twitter when crisis severity increases, as in the outbreak of the H1N1 flu pandemic in 2009 (Chew and Eysenbach, 2010). Xiao et al. (2018) found that humor works differently in two stages of a rumor. They found that humor decreases the perceived severity of a crisis when the rumor is not confirmed but reduces a spokesperson’s sincerity when the rumor is confirmed. However, the mechanism by which the effect of humorous framing varies with the crisis stage remains unknown.

**Figure 1:**
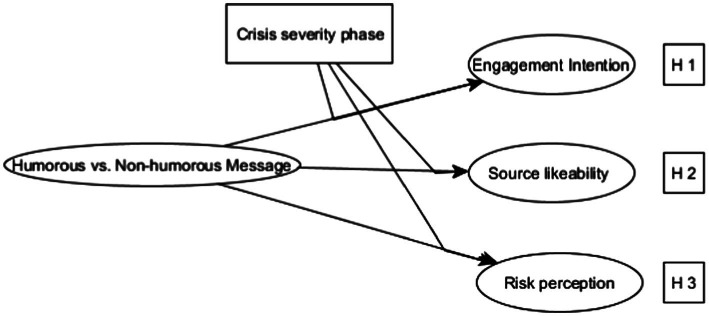
Hypothesized model of the effectiveness of message framing on individuals’ engagement intention, source likability, and risk perception.

To address this question, this research also investigates the interaction effect of humorous appeal and crisis severity on source likeability and perceived risk (see Figure 1). Unlike the CDC’s “Zombie Apocalypse” campaign, the current research explores the effectiveness of a humorous message in communicating social distancing in two phases of crisis severity (low vs. high severity) and evaluates how humor affects an individual’s online and offline engagement intentions, source likeability, and perceived risk during the COVID-19 pandemic. This will also help suggest how humorous framing can be used as an effective crisis communication strategy on social media.

## Theoretical Framework and Hypotheses

RAMS aims to explain the process of media influence on risk perceptions among the general public. Based on Risk Amplification through Media Spread Framework (Vijaykumar et al., 2015), a risk event is defined as a real or perceived threat that poses to the public’s health once a public health community confirmed an infectious disease case or outbreak (IDO) has the potential to spread through a social system. According to Vijaykumar et al. (2015), IDO information can influence the amplification, attenuation, or maintenance of the public’s risk perceptions, and in general, contains fact-based or opinion messages about any scientific, social, physical, or mental aspect of an infectious disease. Instead of the linear risk amplification process of Social Amplification of Risk Framework (SARF), RAMS demonstrates the complexity of using different media channels (face-to-face, traditional media, online media, and social media) in promoting and disseminating IDO information to different targeted audiences (individuals who exposed to an infectious disease, the local population or community, and the broader public), revealing the dynamics of the risk amplification process in the current media landscape (Jin et al., 2018). RAMS also highlights the role of social media in diffusing IDO information through its multimodal nature and “going viral” magic that enables instantaneous sharing of messages through online social networks.

Additionally, RAMS divides four stages of an IDO, from preparedness, initial case(s), increasing number of cases, and “outbreak” (many cases in many places) to “recovery” (significant decrease in the number of cases). Vijaykumar et al. (2015) provide tailored communication strategies according to different IDO stages, for instance providing background information on the disease and its transmission for traditional media, while proving accurate information as soon as possible for online media in the phase of the initial case. However, the recommendations for communication priorities only focus on what to communicate for an online and offline media channel, little attention has been paid to how to make the IDO information visible or “going viral” during the stages of an infectious disease outbreak.

Social media has served as a key source for diffusing time-sensitive information during the COVID-19 pandemic. Research shows more than 86% of African countries’ national health ministries disseminated COVID-19-related information through their social media accounts (Asubiaro et al., 2021). Ohme et al. (2020) found during the first weeks of the COVID-19 pandemic, people in Belgium spent 74% more time than usual on social media apps to stay informed, in sync, and in touch with society. Messages on social media platforms rapidly reach the public and connect people to their broader social networks, while humorous framing helps messages further disseminate at a large scale through thousands of online sharing and liking behaviors (Fraustino and Ma, 2015). Papapicco and Mininni (2020) found humor on social media may be a strategy of commitment in maintaining preventive behavior through its specific communication function of “emotion sharing” in the context of Ph.D. memes. Vicari and Murru (2020) revealed that social media in Italy thrived with humorous content during the early weeks of the COVID-19 pandemic in that country, through memes, multimedia remixes, and jokes. However, the humorous content was mostly in the form of traditional political satire, mocking people in Northern Italy and China, not offering information on appropriate preventive behaviors. The different timing of the pandemic’s peak in geographically distinct locations creates a short window of response opportunity (Nesbitt et al., 2020). If healthcare officials can rapidly disseminate humorous information on preventive behavior instead of mere satirical content about early affected areas to later ones, it may improve the situation in areas affected later.

### The Moderation Role of Severity IDO Phase on Citizen’s Engagement Intention

A humorous message can be defined as a message intentionally, semantically, or structurally manipulated in relation to humorous elements to evoke amusement for both sender and receiver (Speck, 1991; Martin et al., 2003). A message can be perceived as humorous based on shared sets of social norms and knowledge (Meyer, 2000). A case study from China revealed that humorous crisis communication may be particularly valuable on social media—a platform known as interpersonal and less serious and informal than some others (Kim et al., 2016). Kim et al. (2016) found a self-mockery humorous message strategy employed by Alibaba effectively lessened the bad effects of a false advertising scandal benefiting from its informal language tricks on social media. Xiao et al. (2018) also confirmed the effectiveness of humor on social media in decreasing perceived crisis severity during the unconfirmed rumor stage, though they also noted humor might not a good choice when the rumor was confirmed. In the context of crisis communication regarding infectious disease, we expect that the effectiveness of humor in communicating social distancing may also differ in different phases of crisis severity.

Crisis severity is determined by objective criteria related to the event, such as the number of victims, number of injuries, and physical damage (Laufer et al., 2005). A substantial body of research has found a positive association between crisis severity and the public’s attribution of responsibility to the organization involved (Hwang and Cameron, 2008). Coombs and Holladay (1996) suggest that the more severe a crisis is, the more accommodative a response strategy an organization should use. Previous research has shown that in a severe crisis, the public prefers more rational messages that highlight factual information, regardless of the framing style (Xiao et al., 2018, 2019)—consumers in this phase may care more about informative and useful content (Claeys et al., 2013). Thus, we expect humor may be effective in promoting social distancing in low severity IDO phases (e.g., preparedness and recovery phases), while in high severity phases (e.g., outbreak phase), a non-humorous message should work better.

*H1*: For the low severity IDO phase (vs. high severity IDO phase), humorous messages will lead to higher online and offline engagement intention. However, for the high severity condition, such difference vanishes.

### The Moderation Role of Severity IDO Phase on Source Likability

Source likability is defined as an affective evaluation linked to a source (Ewoldsen and Fazio, 1992; Roskos-Ewoldsen et al., 2002). For example, a person who says pleasant things may be perceived as likable (Eagly and Chaiken, 1975). The previous literature on advertising gives strong support for increased source likability through the use of humor (Weinberger and Gulas, 1992). Humor is a key dimension of spokes-characters’ likability (Callcott and Phillips, 1996). Humorous messages also enhance ad likability and brand likability (Speck, 1991), and individuals’ likability toward a scientist can obviously increases when they perceive a scientific message as more humorous (Yeo et al., 2020). Strick et al. (2012) suggest that humor can break resistance to influence because humor can impede the development of negative associations and create positive associations through positive emotional responses (*via* emotional conditioning or feelings transfer).

However, we expect the severity phase will moderate the effect of humor on source likability. For example, an individual’s online engagement toward humorous content tends to decrease on social media when a pandemic becomes an outbreak situation (Chew and Eysenbach, 2010). In the context of crisis communication, when the severity is high (e.g., high death toll/rate), individuals’ latitude of acceptance is likely to be narrow. Therefore, people will pay more attention to key factual information about the crisis (e.g., how fast the disease spreads). Furthermore, a higher level of severity tends to associate with more victims, injuries, and deaths. As a result, humor may not be considered to be appropriate in a severe phase of the crisis. However, when the severity is low, people are more likely to associate humor with the trait of the spokesperson and transfer positive emotional responses to the spokesperson. People tend to follow advice from those they like. Thus, source likability can increase persuasion power by serving as a cue for judgment (Ewoldsen and Fazio, 1992).

*H2*: For the low severity IDO phase (vs. high severity IDO phase), humorous messages will lead to individual’s higher source likability. However, for the high severity condition, such difference vanishes.

### The Moderation Role of Severity IDO Phase on Risk Perception

Apart from source likability, we expect humor works through another route: perceived risk. More specifically, we propose individual’s risk assessment of an infectious disease outbreak (IDO) can be affected by the IDO information framed humorously or not. A survey study conducted in India showed traditional media have tended to calm the public down by broadcasting positive news during the COVID-19 pandemic, while the content on social media platforms has tended to make individuals more fearful (Musa et al., 2020). Mass media now works as a “social amplification station” to shape the public’s perception of risk by either amplifying or attenuating public risk perception (Kasperson et al., 1988). According to the Risk Amplification through Media Spread Framework (RAMS), messages go viral or not based on a range of message characteristics, including IDO information’s valence and ability to evoke an individual’s positive or negative arousal, information virality can indirectly affect social conversations and in the process, shape publics’ risk beliefs and perceptions of the disease. This means that not only what the media says matters, but how they frame risk issues also affects the public’s sense-making of events or subsequent behaviors (Oh et al., 2020). For instance, humorously framed announcement message decreases individuals’ perceptions of climate change risk by reducing anger and fear (Skurka et al., 2018), while fear-arousing sensational Facebook messages led to more user engagement *via* enhanced risk perception during the 2016 Zika virus outbreak (Ali et al., 2019). Oh et al. (2020) revealed social media use during the MERS outbreak can elicit higher individuals’ anger and fear, resulting in enhanced risk perception and more preventive behaviors. However, when crisis severity is high, communicating crisis in a humorous way may leave an impression that the situation is not very serious, because the playful manipulation of humor may function as a psychological coping strategy, temporarily distracting individuals’ attention from the fear of pandemic’s outbreak to amusement, leading them to interpret the risk as less severe (Meyer, 2000). Therefore, we expect the use of humor on social media in the high severity IDO phase (e.g., Outbreak phase of an infectious disease) will lead to lower perceived risk.

*H3*: For the high severity IDO phase (vs. low severity IDO phase), humorous messages will lead to individual’s lower level of perceived risk. However, for the low severity condition, such difference vanishes.

## Materials and Methods

### Recruitment

An experiment was designed to test the research questions during the COVID-19 crisis in China from January 30 to February 2, 2020. The experiment employed a 2 (message framing: humorous vs. non-humorous) × 2 (crisis severity phase: low vs. high) between-subjects design. We recruited participants using the SoJump online sample panel.

### Stimuli and Procedure

For the manipulation of the crisis severity IDO phase, we used a news report about a video going viral on the TikTok video-sharing platform. The video was from the party secretary of a fictional small village with a population of 600; the content was his audiotaped speech communicating the need for social distancing and appealing to everyone to stay at home (screenshots and scripts of the video news see Supplementary Material). For the low-level crisis severity phase, the video reported no confirmed COVID-19 case was found yesterday in that village (“preparedness phase,” see Vijaykumar et al., 2015), while the video for the high severity phase reported 49 COVID-19 cases increased compared to the previous day in that village (“outbreak phase,” many cases continuously reported, see Vijaykumar et al., 2015).

For the manipulation of the message frame, we used two videos (screenshots and scripts of the video see Supplementary Material) adapted from the initial (fictional) post on the TikTok video-sharing platform. The videos were edited to the same length and re-recorded using a Henan dialect accent (an accent that can be understood by people who speak Chinese). Furthermore, a picture of a loudspeaker was displayed as the background, and the same type of subtitle of the audiotape was presented in both videos.

To manipulate the level of humor, we applied the affective humor mechanism (arousal-safety). This humor mechanism is defined as a break from emotional strain, creating the perception that the message is funny (Rothbart, 1977). In the humorous video, the spokesperson used aggressive humor to denigrate people who attempted to go outdoors and gather in groups, then asked everyone to stay at home. In the non-humorous video, the spokesperson appealed to everyone to stay at home through a factual message (see Supplementary Material for scripts).

For the pre-test, we recruited 88 participants (72% males, Mean age = 27.20 years, SD = 5.54) from the SoJump online sample panel. The test suggested successful manipulation of message framing; participants in the humorous message condition (M = 5.26, SD = 1.39) rated the message more humorous than those in the non-humorous message condition [M = 3.73, SD = 1.41; *t* (86) = 5.04, *p* < 0.001]. Participants in the non-humorous message condition (M = 4.84, SD = 1.34) rated the message more rational than the humorous message condition [M = 3.16, SD = 1.26; *t* (86) = 6.02, *p* < 0.001].

For the manipulation of crisis severity phase, participants in the high severity phase (M = 5.35, SD = 1.38) rated the situation as more severe than those in the low severity phase [M = 3.04, SD = 1.42; *t* (86) = 7.64, *p* < 0.001].

All participants answered manipulation check questions and then completed the rest of the questions (e.g., gender and age). The whole procedure took about 5 min.

### Measures

To check the message framing manipulation, an established four-item 7-degree scale of perceived humor (funny/humorous/amusing/entertaining, Cronbach’s *α* = 0.85; Nabi et al., 2007) and a two-item 7-degree scale of perceived rationalness (serious/rational, Cronbach’s *α* = 0.83) were tested. The manipulation of crisis severity was measured using a two-item 7-degree scale from Arpan and Pompper (2003) which asked “how severe/serious do you consider the bad effects caused by the virus in the village to be?” (Cronbach’s *α* = 0.93).

Online engagement intentions are the public’s willingness to participate in public affairs through computer-mediated actions, including positive E-Word-of-Mouth and their intentions to spread the message online (e.g., like, repost, forward, and comment, see Chen et al., 2020), while offline engagement intention is the public’s tendency to take actual protective actions and face-to-face communication (Fraustino and Ma, 2015). The online engagement intention was measured using a three-item 7-degree scale that asked “to what extent will you like the video/forward (retweet) it to your family and friends/leave a positive message after watching the video?” (Cronbach’s *α* = 0.94). Offline engagement intention was measured using a three-item 7-degree scale that asked “to what extent will you follow the quarantine rules/stay at home and not hang out/persuade your family and friends to follow the quarantine rules offline?” (adopted from Fraustino and Ma, 2015, Cronbach’s *α* = 0.79).

Source likeability toward the spokesperson was measured using a three-item 7-degree scale that asked to what extent the participant agreed, “I have a good feeling about the spokesperson/I think the spokesperson has a good overall reputation/The spokesperson is likable” (Cronbach’s *α* = 0.90), based on an emotional evaluation for organizations from Arpan and Pompper (Ponzi et al., 2011).

Risk perception of the virus was measured using a four-item 7-degree scale that asked “to what extent do you think the COVID-19 virus is dangerous to yourself/the probability that I will get infected the COVID-19 disease is high/the COVID-19 pandemic situation is quite severe/there will be an outbreak of the virus in the near future?” (Cronbach’s *α* = 0.75).

The perceived threat of the COVID-19 situation in an individual’s city of residence was measured with a 7-degree scale by asking to what extent do you think the severity of the COVID-19 situation in your city of residence.

### Procedure

Participants were asked to rate the perceived threat of the COVID-19 situation in the city of residence and then randomly assigned to one of four conditions. The video news report about the COVID-19 situation (high vs. low severity phase) in the village was presented first, followed by an attentional multiple-choice question asking how many confirmed cases were reported in the village. Participants who gave the wrong answer to this question were automatically excluded (*n* = 38).

Then, another video (with humorous vs. non-humorous message) was presented, followed by an attention filter (a multiple-choice question) asking for the exact code presented at the very end of each video to ensure participants watched the video. Participants who gave the wrong answer were automatically excluded (*n* = 23). This was followed by a multiple-choice question asking if they had watched the video clip before; participants who responded “yes” were automatically excluded (*n* = 0).

A total of 139 valid responses were collected (48% males, Mean age = 27.27, SD = 5.80).

## Results

### Manipulation Checks

Testing suggested a successful manipulation of message framing; participants in the humorous message condition (*M* = 5.09, *SD* = 1.29) rated the message as more humorous than those in the rational message condition [*M* = 3.98, *SD* = 1.53; *t* (137) = 4.64, *p* < 0.001]. Meanwhile, participants in the rational message condition (*M* = 4.81, *SD* = 1.29) rated the message more rational than the humorous message condition [*M* = 3.12, *SD* = 1.13; *t* (137) = 8.17, *p* < 0.001].

For the manipulation of crisis severity, participants in the high severity condition (*M* = 5.17, *SD* = 1.45) rated the situation of that village more severe than those in the low severity condition [*M* = 3.01, *SD* = 1.87; *t* (137) = 7.51, *p* < 0.001].

### Correlation Analysis

Risk perception and source likeability are both positively correlated with an individual’s engagement intention (Table 1). This implies that when participants have a higher perception of the risk of the disease and feel the spokesperson is more likable, they are more likely to engage with the message.

**Table 1:** Correlation matrix and descriptive statistics.

Measures	Risk perception	Source likeability	Offline engagement	Online engagement	*M*	*SD*
Risk perception	/	0.32^**^	0.46^**^	0.49^**^	5.15	1.47
Source likeability	0.32^**^	/	0.37^**^	0.44^**^	5.33	1.24
Offline engagement	0.46^**^	0.37^**^	/	0.49^**^	5.66	1.38
Online engagement	0.49^**^	0.44^**^	0.49^**^	/	4.73	1.38

** *Correlation significant at the 0.01 level*.

### Main Effects

A *t*-test was conducted to test the main effect of humorous framing on online engagement intention; the results revealed no significant difference between a humorous message (*M* = 4.80, *SD* = 1.33) and a rational one [*M* = 4.66, *SD* = 1.44; *t*(137) = 0.61, *p* = 0.49]. Another *t*-test tested the main effect of humorous framing on offline engagement intention; the results revealed no significant differences between the humorous message (*M* = 5.59, *SD* = 1.48) and rational message [*M* = 5.73, *SD* = 1.29; *t* (137) = 0.59, *p* = 0.34].

An additional t-test was tested the main effect of humorous framing on source likability; the results revealed a significant difference between a humorous message (*M* = 5.55, *SD* = 1.22) and a rational one [*M* = 5.10, *SD* = 1.23; *t*(137) = 2.18, *p* = 0.03]. Another *t*-test tested the main effect of humorous framing on risk perception; the results revealed a significant differences between the humorous message (*M* = 4.84, *SD* = 1.47) and rational message [*M* = 5.46, *SD* = 1.39; *t* (137) = 2.56, *p* = 0.01].

### Moderation Effects

An ANOVA analysis was conducted to test the moderation effect of humorous framing and IDO severity phase on online and offline engagement intentions controlling for participants’ gender and age; the results revealed a significant moderation effect of a humorous message and severity phase on online engagement intention [*F* (1,133) = 6.93, *p =* 0.009, *η*^2^ = 0.052, power = 0.79] and also a marginal significant moderation effect of a humorous message and severity phase on offline engagement intention [*F* (1,133) = 3.08, *p* = 0.082, *η*^2^ = 0.023, power = 0.43].

**Figure 2:**
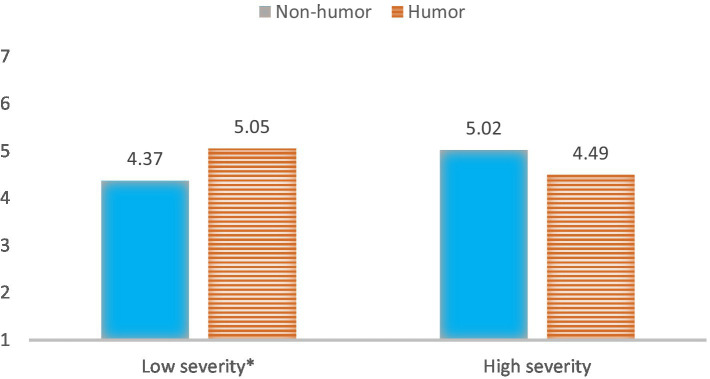
Interaction of message framing and crisis severity phase on online engagement intention.

More specifically, a Post-Hoc analysis showed that using humor (M = 5.05 SD = 0.21) led to more online engagement intention than the non-humorous message (M = 4.37 SD = 0.22) in a low-level crisis severity phase (*p* = 0.03). However, when participants were told the video was from a high severity crisis scenario, no significant difference was found between a humorous and non-humorous message online engagement intention (*p* = 0.12, see Figure 2).

**Figure 3:**
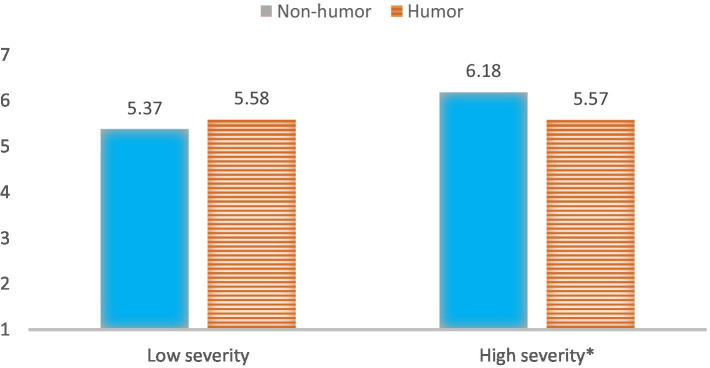
Interaction of message framing and crisis severity phase on offline engagement intention.

However, using humor (M = 5.57, SD = 0.24) led to less offline engagement intention than the non-humorous message (M = 6.18 SD = 0.24) in a high-level crisis severity phase (*p* = 0.08). However, when participants were told the video was from a low severity crisis scenario, no significant difference was found (*p* = 0.50, see Figure 3).

**Figure 4:**
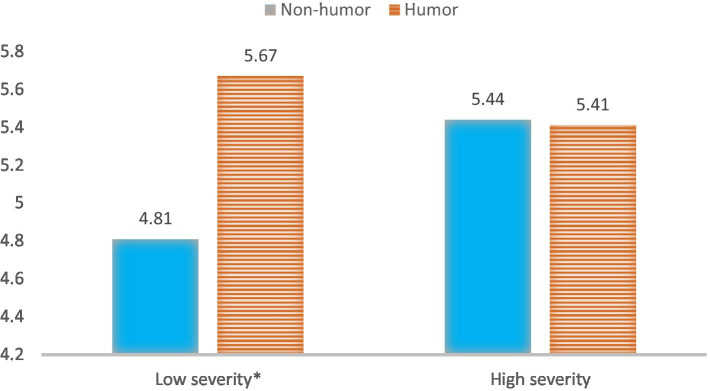
Interaction of message framing and crisis severity phase on source likability.

Additionally, an ANOVA analysis was also conducted to test the moderation effect of humorous framing and IDO severity phase on source likability controlling for participants’ gender and age. The results revealed a significant moderation effect of a humorous message and severity phase on source likeability [*F* (1,133) = 4.38, *p =* 0.004, *η*^2^ = 0.032, power = 0.57, see Figure 4]. Specifically, the humorous message (M = 5.67, SD = 0.20) led to higher source likeability than the non-humorous message (M = 54.81, SD = 0.20) in a low severity condition (*p* < 0.01, see Figure 4). However, in the high severity condition, no significant difference in offline engagement was found between a humorous and a non-humorous message on source likability (*p* = 0.93).

**Figure 5:**
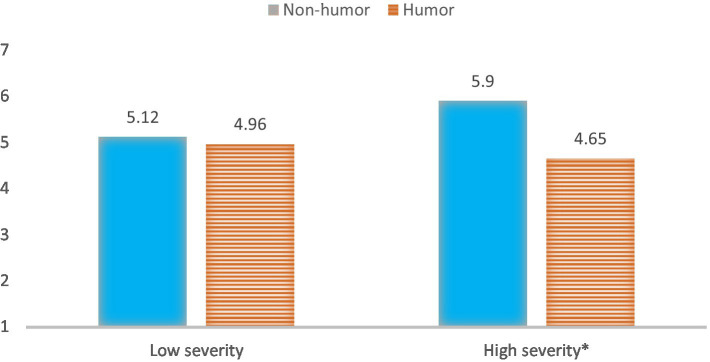
Interaction of message framing and crisis severity phase on risk perception.

And also a significant moderation effect of a humorous message and severity phase on perceived risk [*F* (1,133) = 5.45, *p* = 0.021, *η*^2^ = 0.039, power = 0.65, see Figure 5]. The test suggested that the humorous message (M = 4.65, SD = 0.24) decreased perceived risk more than the non-humorous message (M = 5.90, SD = 0.24) in a high severity condition (*p* < 0.001). However, in the low-level crisis severity condition, we observed no significant effect of a humorous (vs. non-humorous) message on perceived risk (*p* = 0.61).

## Discussion

### Principal Results

The current study revealed the effectiveness of using humor on social media to communicate the need for social distancing for infectious disease, and how the effects of humor are moderated by the phase of the crisis. The results of this experimental study conducted during the first weeks of the COVID-19 pandemic in China demonstrated that the severity of the current phase of a health crisis can significantly affect stakeholders’ online and offline responses to the disease on social media.

More specifically, in a low severity phase, a humorous (vs. non-humorous) message leads to increased individual’s online intentions, and no significant interaction effect was found in a high severity phase. Whereas in a high severity phase, humor reduced individual’s offline engagement intentions. Through a decrease in perceived risk, no significant interaction effect was found in a low severity phase, which means H1 is partially supported.

Additionally, in a low severity phase, a humorous (vs. non-humorous) message leads to increased source likability toward the message, and no significant interaction effect was found in a high severity phase (H2 supported). Whereas in a high severity phase, humor reduced individual’s perceived risk, and no significant interaction effect was found in a low severity phase (H3 supported).

### Theoretical Contributions

The current research makes several contributions to the research on the use of humor in crisis and risk communication. First, we considered the dynamics of the infectious disease severity phase when discussing the use of humor in communicating about an infectious disease. The results illustrate the boundary conditions for the effects of a humorous message on individuals’ online and offline engagement. The CDC’s “Zombie Apocalypse” campaign on social media showed humor only has limited effectiveness in spurring online engagement such as liking and sharing behaviors to help quickly spread the medical information, and it did nothing to help motivate more protective action offline (Fraustino and Ma, 2015), while Skurka et al. (2018) demonstrated that a humorous video can help to produce greater offline climate change mitigation behavioral intentions through increased perceived humorousness. We revealed that in line with Fraustino and Ma (2015), humor can motivate both more online engagement intention, however, it works only when the crisis severity phase is low. When crisis severity soars, a humorous message would decrease an individual’s offline protective action. This is consistent with previous research that suggests humor is more desirable in crisis communication when the crisis is not severe (Vigsø, 2013; Kim et al., 2016; Xiao et al., 2018). When the crisis becomes severe, the public prefers objective facts and information, rather than emotion-arousal manipulations of the message (Claeys et al., 2013; Xiao et al., 2018, 2019). Going beyond previous research, this study recognized that crisis severity changes in different phases of the spread of an infectious disease (Jin et al., 2018), thereby providing actionable strategy selections for crisis practitioners in a dynamic communication environment.

Second, we revealed using humor in communicating information about an infectious disease can enhance the spokesperson’s likeability in a low severity phase. This is in line with the results of past research that individuals tend to associate peripheral humor with the spokesperson and transfer positive emotional responses to the spokesperson when they are only marginally involved in an event (Zhang and Zinkhan, 1991). Social media channels are known to be a relatively interpersonal and informal mode that provides a more natural context to speak with a conversational human voice (Kelleher, 2009). Humorous responses may confer more likeability because presenting the information in a playful manner shows the public a more human side of an organization.

Third, the negative side of using humor in communicating an infectious disease appears in severe crisis phases, as it then decreased the public’s perception of risk. This confirms the findings of Skurka et al. (2018) that humorous public announcement messages decrease individuals’ perceived risk through reduced anger and fear. Unfortunately, individuals’ anger and fear are essential in increasing risk perception and preventive behaviors (Oh et al., 2020). Therefore, the relief function of humor plays an undesirable role in this situation, and a non-humorous message without any emotion-arousal manipulation is more favorable for this period.

### Managerial Implications

The findings of this research also have several managerial implications. According to the RAMS model, specific response planning and communication priorities should be integrated based on the current phase of an infectious disease (Vijaykumar et al., 2015; Jin et al., 2018). There is the potential to fruitfully use humor in the preparedness and recovery phases of an infectious disease outbreak. The different timing of the peak of a widespread pandemic in geographically distinct locations creates a short window of response opportunity for late-affected areas (Nesbitt et al., 2020). For those areas that are in a less severe stage, humor may help to spread the message regarding the correct protective action rapidly and thus save lives. Similarly, after the outbreak of the disease, humor may help to remind the public how to live with the virus in the recovery phase. Public health information officers and communication practitioners need to timely communicate the accurate IDO information and meanwhile be prepared with framing strategies that can help the information widely spread at each IDO phase.

In addition, scientists and professionals working in health departments (e.g., CDC and state health department) face limits when an infectious disease hits (Jin et al., 2018). Scientists and experts have found that using humor can help them to elicit more engagement through enhancing perceived expertise, but not likeability (Yeo et al., 2020). However, based on this research, non-professionals (e.g., mayors, village/community leaders, and popstars) can potentially use humor for likeability and use that to call for more effective engagement in a low severity phase.

Third, as social media continues to play an increasingly important role as a “social amplification station” to shape the public’s perception of risk (Kasperson et al., 1988), humor should be used cautiously when the risk threat of a crisis event must be amplified—for instance when an infectious disease outbreak becomes severe. In such cases, the provision of objective facts without a humorous slant should be more favorable.

### Limitations

Although this study provides both theoretical and practical implications on the effectiveness of humor in communicating an infectious disease, it is not without limitations.

Firstly, we tested only one humorous framing style (aggressive humor) and one intensity level of humor in our stimuli. Given that different humor framing styles and intensities may affect the effectiveness of a message (Meyer, 2000), future research should examine more humor styles (e.g., self-deprecating) and compare the effects of different intensities of humor.

We also conducted the experiment during the COVID-19 pandemic, so individuals’ actual experience of the risk in different places may have affected their sense-making of the humorous message. Future research should conduct a field experiment and include more participants from different places with different risk threat levels, and discuss how different risk levels affect the effectiveness of humor in communicating about an infectious disease.

Thirdly, in this study, we manipulated severity by presenting different numbers of cases, which was not in accordance with previous severity manipulations (e.g., Xiao et al., 2018). Note that this study was conducted 2 weeks after the outbreak in Wuhan. At that time, the number of cases could suggest how serious the situation was. However, the number of cases can also imply susceptibility. Therefore, future studies may manipulate severity in different ways to if such effects exist when susceptibility is controlled for.

Last but not least, the sample size of this study is relatively small. This may result in increases the likelihood of a Type II error and produce inconclusive results. Therefore, this study may serve as a pilot study to examine the effect of humor in health-related crisis communication. Future studies are needed to see if the findings obtained from the current study could be replicated.

## Data Availability Statement

The raw data supporting the conclusions of this article will be made available by the authors, without undue reservation.

## Ethics Statement

The studies involving human participants were reviewed and approved by Tianjing University. The patients/participants provided their written informed consent to participate in this study.

## Author Contributions

YX and SY contributed to conception and design of the study and wrote the manuscript together. All authors contributed to the article and approved the submitted version.

## Funding

This work was supported by Tianjin University (no. 2021XSC-0092) and the China Postdoctoral Science Foundation (no. 2019M660696).

## Conflict of Interest

The authors declare that the research was conducted in the absence of any commercial or financial relationships that could be construed as a potential conflict of interest.

## Publisher’s Note

All claims expressed in this article are solely those of the authors and do not necessarily represent those of their affiliated organizations, or those of the publisher, the editors and the reviewers. Any product that may be evaluated in this article, or claim that may be made by its manufacturer, is not guaranteed or endorsed by the publisher.
