# Supplementary material for: Using Humor to Promote Social Distancing on Tiktok During the COVID-19 Pandemic
Source: Front Psychol. 2022 Jun 23;13:887744. doi: 10.3389/fpsyg.2022.887744 (PMC9261496; doi:10.3389/fpsyg.2022.887744)
Supplement: Supplementary file 1 [file Data_Sheet_1.docx]

**Appendix A**. **Scripts of video news report of the COVID-19 situation in the village: low severity vs. high severity crisis phase.**

**Low severity crisis phase:**

**The village leader’s loudspeaker warning became a national hit on TikTok**

**[Image of the loudspeaker]**

Zhongshan village’s party secretary loudspeaker warning micro-video became a national hit on TikTok. The content was his audiotaped speech communicating the need for social distancing and appealing to every citizen to stay at home. Zhongshan village has a population of 600 in Henan Province—until yesterday, no case of COVID-19 was found in the village.

**High severity crisis phase:**

**The village leader’s loudspeaker warning became a national hit on TikTok**

**[Image of the loudspeaker]**

Zhongshan village’s party secretary loudspeaker warning micro-video became a national hit on TikTok. The content was his audiotaped speech communicating the need for social distancing and appealing to every citizen to stay at home. Zhongshan village has a population of 600 in Henan Province—until yesterday, 49 cases of COVID-19 increased in the village.

**Appendix B**. **Audiotape scripts of the village leader’s loudspeaker warning in the TikTok video clip**

**Humorous message:**

No matter whether your car number begins with ABCDEFG (Pronounced in Chinese Pinyin) or HIJKLMN. No matter if your car is a Benz, BMW, PASSAT, JETTA, LADA (which doesn’t actually exist), or MAZDA. Better stay home safe instead of showing off your car around!

**Non-humorous message:**

To combat the COVID-19 virus, everyone should stay home and stop all outdoor activities. Remember a facial mask if you have to go shopping for necessities. For the sake of your health and that of others, keep your door closed and stay home safe!
